# CovidCounties is an interactive real time tracker of the COVID19 pandemic at the level of US counties

**DOI:** 10.1038/s41597-020-00731-8

**Published:** 2020-11-16

**Authors:** Douglas Arneson, Matthew Elliott, Arman Mosenia, Boris Oskotsky, Samuel Solodar, Rohit Vashisht, Travis Zack, Paul Bleicher, Atul J. Butte, Vivek A. Rudrapatna

**Affiliations:** 1 Bakar Computational Health Sciences Institute, University of California, San Francisco, San Francisco, CA USA; 2 School of Medicine, University of California, San Francisco, San Francisco, CA USA; 3 Department of Medicine, University of California, San Francisco, San Francisco, CA USA; 4 Evident Health Strategies, West Newton, MA USA; 5 Department of Pediatrics, University of California, San Francisco, San Francisco, CA USA; 6 Center for Data-Driven Insights and Innovation, University of California Health, Oakland, CA USA; 7 Division of Gastroenterology, Department of Medicine, University of California, San Francisco, San Francisco, CA USA

**Keywords:** Viral infection, Epidemiology

## Abstract

Management of the COVID-19 pandemic has proven to be a significant challenge to policy makers. This is in large part due to uneven reporting and the absence of open-access visualization tools to present local trends and infer healthcare needs. Here we report the development of CovidCounties.org, an interactive web application that depicts daily disease trends at the level of US counties using time series plots and maps. This application is accompanied by a manually curated dataset that catalogs all major public policy actions made at the state-level, as well as technical validation of the primary data. Finally, the underlying code for the site is also provided as open source, enabling others to validate and learn from this work.

## Introduction

The disease known as COVID-19 was first reported in December of 2019 in Wuhan, China^1^. Three months later it was declared a pandemic by the WHO, and since then its death toll has reached over 820,000 while infecting over 24 million people across 210 countries worldwide^2^. Additionally, the pandemic has disrupted the daily lives of billions and has incurred significant socioeconomic costs at the global level.

In the US, the very assessment of the disease’s impact has been challenged by limitations in accurate data capture and analysis. Variable testing, uneven reporting, barriers to data sharing, and a lack of easy-to-use analytic tools have all contributed to a lack of clarity in establishing and trending the state of the pandemic. As a consequence, policy makers at all levels have been forced to make decisions of great socioeconomic consequence in the face of significant uncertainty.

To improve the accessibility of basic COVID-19-related information in the US, especially by the general public and policymakers without a data science background, we report the creation of a new interactive visualization tool that depicts daily disease trends at the level of individual US counties. This web application features the novel reuse of several publicly available sources of data while also introducing a new, manually curated dataset accompanying this manuscript. This site features several unique views, including local doubling times and estimated ICU bed requirements by county. Additionally, we report the technical validation of the primary data (counts per county or state per day) against other official- and commonly used sources of data.

## Results

### Data sources

CovidCounties derives a majority of its data from The New York Times Coronavirus github page [https://github.com/nytimes/covid-19-data] which is updated daily with cases and deaths reported in each state and county from the previous day. This time series dataset was derived from a variety of governmental sources. However, to our knowledge this data has never been formally validated against other reputed sources of COVID-19 reporting including state and local departments of public health.

### Technical validation

**Fig. 1:**
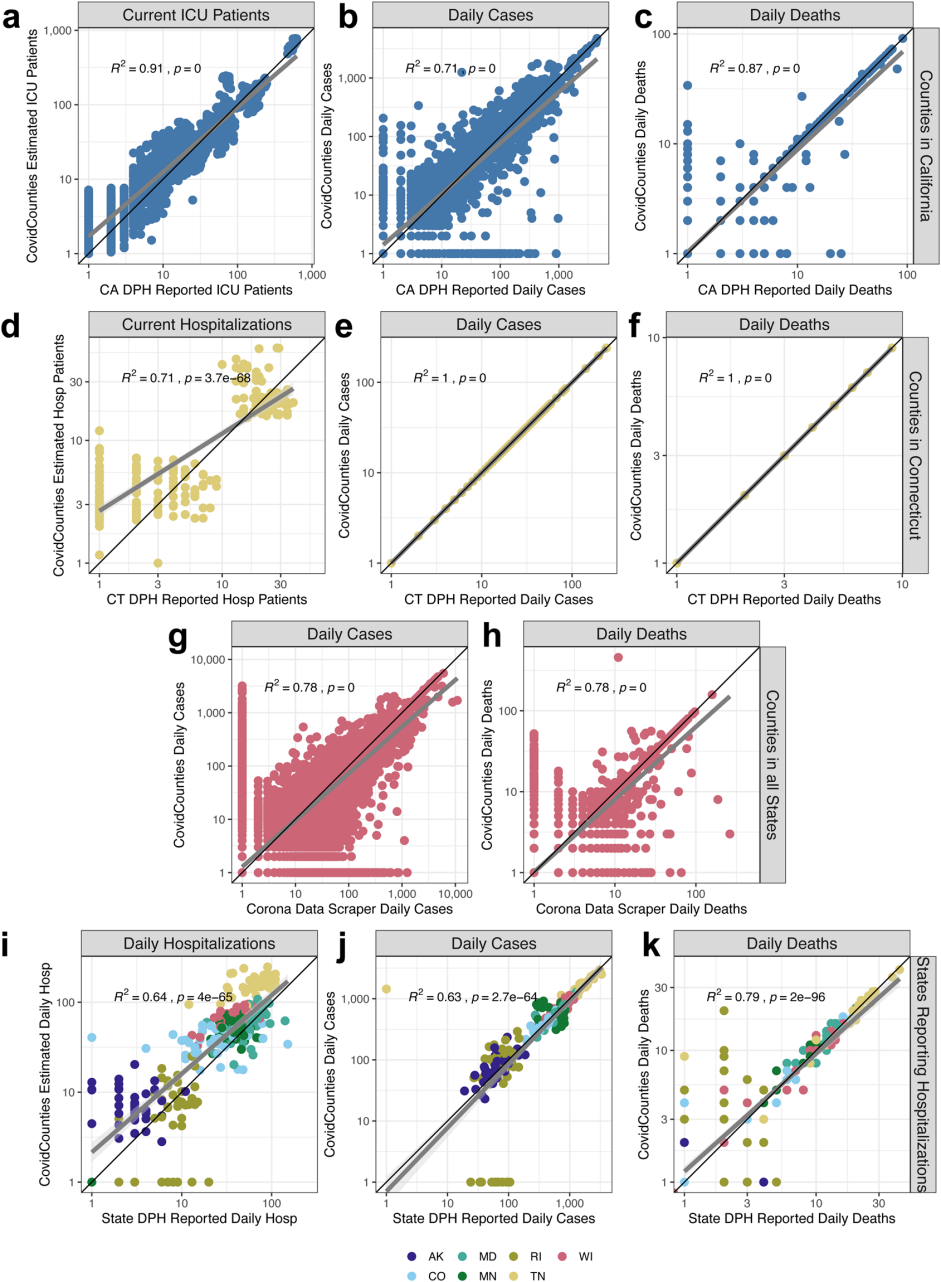
Technical validation of the datasets. (**a**–**c**) Comparison of estimated ICU bed occupancy (n = 2,321) (**a**), daily cases (n = 2,348) (**b**), and daily deaths (n = 2,348) (**c**) reported by CovidCounties against corresponding data reported by the California Department of Public Health. Each point corresponds to a measurement from a given California county on a particular date where both datasets report counts. Data is from 6/28/2020 - 8/8/2020. (**d**–**f**) Comparison of the estimated hospital bed occupancy (n = 248) (**d**), daily cases (n = 240) (**e**), and daily deaths (n = 240) (**f**) reported by CovidCounties against corresponding data reported by the Connecticut Department of Public Health. Each point corresponds to a measurement from a given Connecticut county on a particular date where both datasets report counts. Data is from 6/28/2020 - 8/8/2020. (**g**,**h**) Comparison of the estimated daily cases (n = 121,944) (**g**) and daily deaths (n = 121,944) (**h**) reported by CovidCounties against corresponding data reported by the website Corona Data Scraper. Data is from 6/28/2020 – 8/8/2020. Each point corresponds to a measurement from any US county in the dataset at a particular time where both datasets report counts. (**i**–**k**) Comparison of the estimated hospital bed occupancy (n = 287) (**i**), daily cases (n = 287) (**j**), and daily deaths (n = 287) (**k**) reported by CovidCounties against corresponding data reported by 7 different state Departments of Public Health. Data is from 6/28/2020 – 8/8/2020; curated state data is available in the data file accompanying this manuscript. R^2^ and p-values are derived from the Pearson correlation coefficient.

First, we demonstrate the high concordance of daily cases and deaths calculated and displayed in CovidCounties at the county level by directly comparing these to numbers reported by the Departments of Public Health in California and Connecticut (Fig. 1a–f). These two states were chosen because they both publicly report the daily counts of cases requiring intensive care (Fig. 1a) or hospitalization (Fig. 1d) at the county level. R^2^ rates corresponding to the concordance between CovidCounties countywide reported daily cases/deaths derived from the New York Times data curation efforts and the state reported countywide daily cases/deaths in California and Connecticut ranged from 0.71 to 1. To our knowledge, California is only state in the US to report comprehensive daily county-wide ICU bed utilization rates and Connecticut is one of a few states to report comprehensive daily county-wide hospitalizations. We found a high degree of concordance between the CovidCounties estimated daily ICU patients in each California county with the numbers reported by the California Department of Public Health (R^2^ = 0.91). Similarly, we found a high degree of concordance between the CovidCounties estimated daily hospitalizations in each Connecticut county with the numbers reported by the Connecticut Department of Public health (R^2^ = 0.71). Despite using a relatively simplistic model, we are able to explain a fairly high degree of the data variation with minimal model bias (Fig. 1a,d).

An R^2^ of 1 was specifically found with respect to cumulative cases and deaths in Connecticut (Fig. 1e,f), suggesting a shared common data source between the New York Times curated data and the Connecticut Department of Public Health.

To establish the variability of different reporting sources, we compared the concordance of our New York Times derived data with that reported by Corona Data Scraper [https://coronadatascraper.com/]. The Corona Data Scraper is another widely used source of aggregated publicly-available COVID-19 timeseries data at the county level which is programmatically aggregated as opposed to the massive human component involved in the curation of the New York Times data used in CovidCounties. We found reasonable concordance (R^2^ = 0.78) for daily reported cases and deaths (Fig. 1g,h).

Lastly, we compared the concordance of our predicted daily hospitalizations, daily cases, and daily deaths at the state level derived from the New York Times data against data reported by 7 different State Departments of Public Health with consistent statewide hospitalization reporting (Fig. 1i–k). The concordance between the CovidCounties estimated daily statewide hospitalizations with the numbers reported by 7 State Departments of Public Health was reasonable (R^2^ = 0.64). A similar level of concordance was found between the CovidCounties statewide reported daily cases/deaths derived from the New York Times data and state reported daily cases/deaths in 7 states (R^2^ = 0.63–0.79). Comparison between CovidCounties estimated ICU patients and hospitalizations and State Departments of Public Health (Fig. 1a,d,i) are used to estimate an 80% CI of the reported point estimate ±58.5%.

### Missing data

Descriptive statistics on the New York Times data are provided in Table 1. 24 states reported cases with unknown counties of residence, however, in all states except Rhode Island and Wisconsin these cases made up less than 2% of the total cases in that state (Table 1). The inability to map these cases to specific counties may explain some of the discrepancies between the New York Times data used in CovidCounties and the curated data from state public health departments and the Corona Data Scraper.

**Table 1:** Descriptive statistics on the New York Times data set.

*New York Times*		
*% of counties with non-missing data*^*†*^	99.4%	
*States with greatest % of counties reporting*	40 states tied at 100%*	
*States with lowest % of counties reporting*	Hawaii (80.0%-4/5)	Alaska (89.7%-26/29)
*States with highest % of unknown cases*	Rhode Island (8.3%; 1,738; 1,644)	Wisconsin (6.2%; 4,534; 780)
*Counties with highest cases per million*	Trousdale, Tennessee (144,297)	Lafayette, Florida (129.295)
*Counties with the fastest doubling times*	Dawes, Nebraska (1.57 days)	Sanders, Montana (2.46 days)
*Counties with highest estimated ICU needs*	Madera, California (200%-20/10)	Rosebud, Montana (184%-1.84/1)

Data reported as of 8/20/2020. States with highest % of unknown cases shows the percent of cases from unknown counties as a fraction of total cases in the state, the absolute number of cases from unknown counties in the state, and the cases per million from unknown counties in the state. States with lowest % of counties reporting shows the percentage of counties reporting, the number of counties reporting, and total counties in the state.

^†^In the NY Times data all counties that reported cases also reported deaths (or were assumed to be 0).

^*^AL, AZ, AR, CA, CT, DE, DC, FL, GA, ID, IL, IN, IA, KY LA, ME, MD, MA, MI, MN, MS, MO, NH, NJ, NY, NC, ND, OH, OK, PA, RI, SC, SD, TN, VT, VA, WA, WV, WI, WY.

### Public policy

**Fig. 2:**
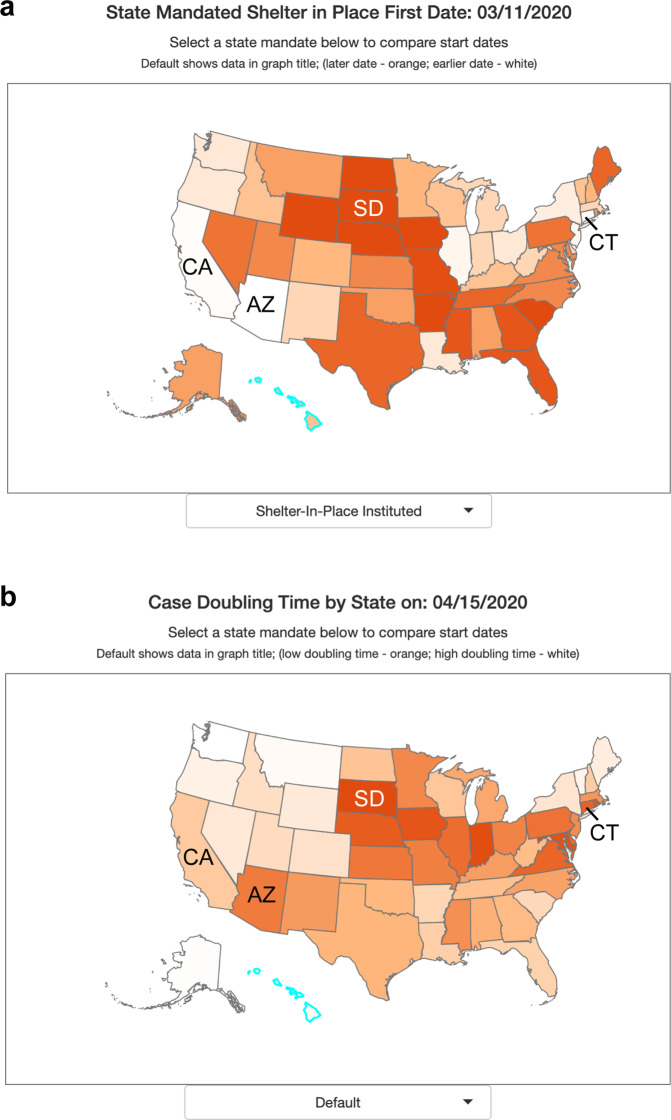
Effect of shelter in place orders on doubling time. (**a**) States within the United States are color-coded by percentile of date to implement state mandated shelter in place. White indicates earlier dates (among states) while dark orange indicates later dates or no state mandate. (**b**) States within the United States are color-coded by percentile of case doubling time on April 15, 2020. Dark orange indicates a fast doubling time (among states), white indicates a slow doubling time.

The data and tools incorporated into CovidCounties support the effectiveness of social distancing measures, consistent with several events that have occurred following the initial release of the website. South Dakota, one of six states which did not have a statewide shelter in place order as of April 15, 2020, experienced rapid case growth following an exposure at a meat plant (Fig. 2a). This accounted for more than half of the state’s cases^3^ as of April 15, 2020, with the fastest statewide doubling time of 4.5 days on April 15, 2020 (Fig. 2b). By contrast, states with early shelter in place times like Arizona on March 11, 2020, California on March 19, 2020, and Connecticut on March 20, 2020 (Fig. 2a) had much slower doubling times of 19.3 days, 22.8 days, and 12.7 days respectively on April 15, 2020 (Fig. 2b).

### Web application deployment

**Fig. 3:**
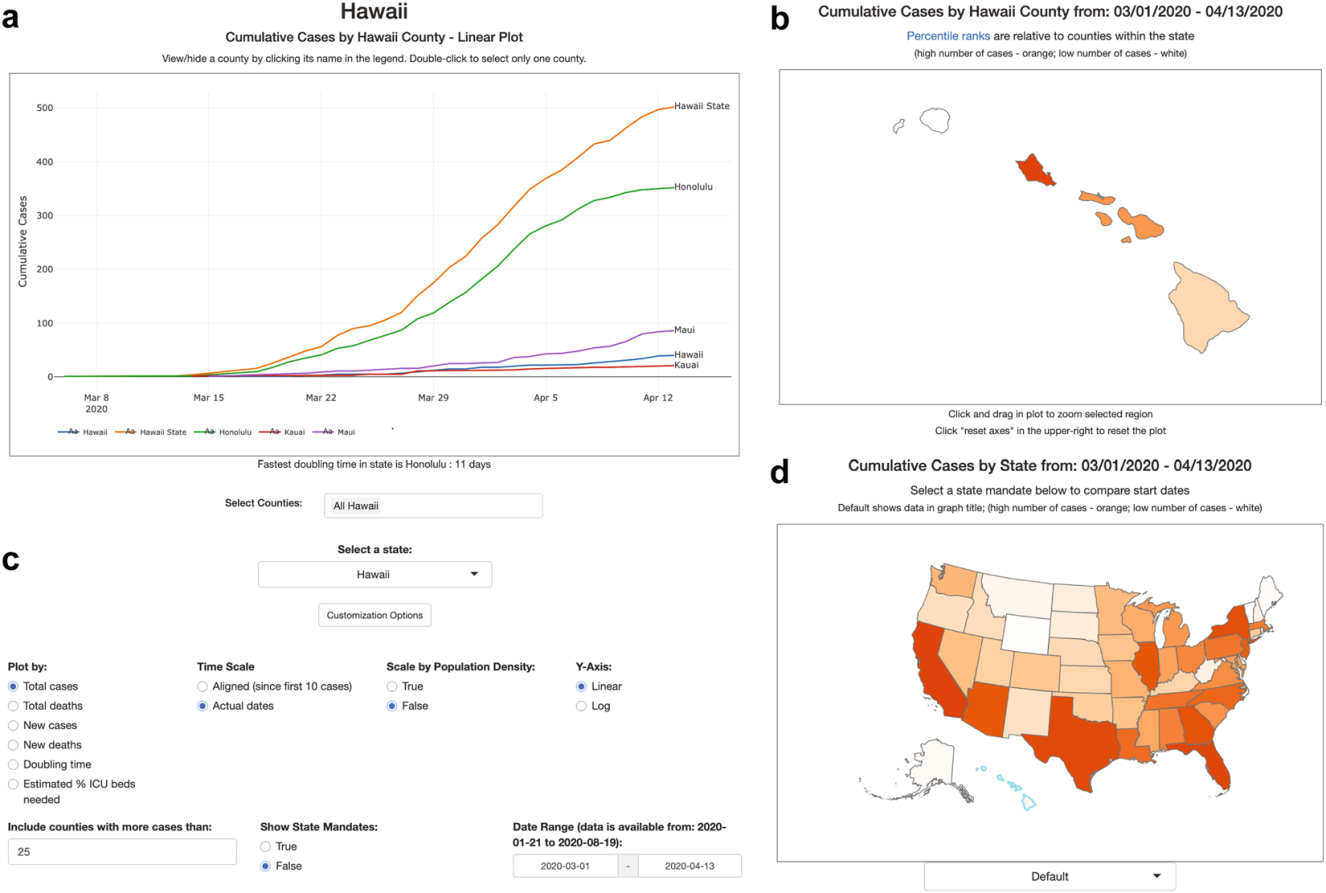
Overview of CovidCounties.org. (**a**) The primary view of CovidCounties.org is the line plot view, depicting time-series trends by individual county. Depicted counties may be selected by single or double clicking the counties displayed in the legend. They may also be selected by typing in counties (including from outside of a given state) at the bottom. (**b**) User-selected individual states are color coded according to the variable of interest (e.g. cumulative cases). Dark orange corresponds to the highest percentiles within the state, white indicates the lowest percentile. Hovering functionality displays statistics corresponding to a given county. (**c**) Line plot views can be extensively customized, with features to enable axis re-centering/scaling, count normalization, depiction of doubling time, and predicted percentage ICU bed utilization. Individual state and United States plots update to reflect selected parameters where appropriate. (**d**) States within the United States are color-coded by percentile according to the variable of interest (e.g. cumulative cases). Dark orange indicates relatively high percentile (among states), white indicates low percentile. Hovering functionality displays statistics corresponding to a given state. The dropdown menu below allows the user to change the view to depict timing that various social distancing policies were implemented: white indicates relatively early adoption (by percentile), dark orange indicates late or no current adoption.

The web application located at covidcounties.org was first released to the public on April 3, 2020. It features two sections: a line plot depicting time-series trends in disease dynamics, and a map depicting geospatial relationships (Fig. 3). The site has had over 15 thousand unique site in the first week and a total of nearly 27 thousand unique site visits over the lifetime of the site, most of whom accessed the website using a mobile device.

## Discussion

The effective management of the COVID-19 pandemic has been hindered by both inaccurate data collection and reporting, as well as relative inaccessibility by non-data scientists. Taken together, these difficulties have impeded optimal policymaking by both government (imposing social distancing policies) and health systems (anticipating ICU utilization) alike. Consequently, responses across institutions have been highly variable and with varying degrees of success. To help address these gaps we developed covidcounties.org and performed the technical validation reported in this work.

The curation of COVID-19 case and death counts by The New York Times is an impressive effort by over 60 reporters to collect, curate and analyze a constantly growing and evolving dataset^4^. However, they acknowledge that the underlying data is extremely fragmented and comes from thousands of different sources at both the state and county levels and thus is inherently limited by accuracy, consistency, and timeliness. The New York Times notes that reported cases have been corrected mere hours after the initial report and there have been numerous instances where data has disappeared from databases without explanation. The New York Times has also chosen to count patients where they were treated rather than their place of residence and report on a number of geographic exceptions in their dataset (https://github.com/nytimes/covid-19-data) including the treatment of cities like New York City and Kansas City and the allocation of cases from cruise ships. Further, there are a subset of cases where the patient’s county of residence cannot or has not yet been identified which is generally a small fraction of a state’s total cases but can be a significant number in a small state like Rhode Island (Table 1).

Taken together, these subtleties of the data collection process imply that the COVID-19 data from The New York Times may not exactly agree with the numbers reported by various state and county Departments of Public Health. We quantified the consistencies between The New York Times COVID-19 data at the county (Fig. 1b,c,e,f) and state (Fig. 1j,k) level using state Department of Public Health data and found the datasets to be largely comparable. Based on the exact agreement, it seems likely that The New York Times is deriving their data for Connecticut directly from the Connecticut Department of Public Health (Fig. 1e,f).

Anomalies in the data become more apparent when comparing different reporting sources. In Fig. 1j,we observe a set of points where the CovidCounties daily cases for the state Rhode Island obtained from the New York Times are zero and the Rhode Island Department of Public Health reports these values to be nonzero. Inspection of the dates corresponding to these values reveals that these all occurred on weekends and that all the cases that occurred over the weekend were attributed to the following Monday. While this would have a small impact on our prediction of current hospitalizations and current ICU patients due to the 10-day average length of stay, this does have a direct impact on daily hospitalizations (Fig. 1i).

Despite the simple model formulation used to estimate ICU patients and hospitalizations and parameters that are derived from averages across multiple states rather than for specific states or counties, the concordance with data reported by State Departments of Public Health is quite reasonable (Fig. 1a,d,i). We would like to highlight that this concordance and variability is similar to the concordance of underlying data used by the model in the form of daily cases reported by the New York Times and the daily cases reported by the States Departments of Public Health (Fig. 1b,j). This variability is due to the inherent noise and error in the reporting and aggregation of this data from a vast number of sources without any specified reporting standards. The true power of using such a simple model is that it is easily extrapolated to any county in the United States on any given date provided the previous 10 days of daily cases are available. While our benchmarking demonstrates the internal validity of our modeling approach, due to a lack of readily available external datasets we are unable to assess the external validity which is a limitation of this approach.

With the advent of the COVID-19 pandemic we have observed a trend towards government agencies at the municipal, county, state, and national levels making their data increasingly accessible for re-use and therefore provide potential value. However, many of the most popular tools which are built upon this freely available data do not provide their source code for further development. The Johns Hopkins dashboard^2^, which receives more than 1.2 billion hits per day, has made their data publicly available^5^, however, the source code for their dashboard is not made available for further development by third parties. Similarly, the IHME dashboard^6^ which has been referenced by the White House for making policy decisions^7^ has had their dashboard peer reviewed^8^, however, their epidemiological model has yet to be peer reviewed^9^. While IHME provides open source code on their data aggregation process (https://github.com/beoutbreakprepared/nCoV2019) and some features of their model including the curve fitting of their projections (https://github.com/ihmeuw-msca/CurveFit), the whole dashboard is not open source. Additionally, many states and counties are using *Tableau*, a proprietary piece of software, to visualize COVID-19^10^ and as of 8/23/2020 there are 27,431 coronavirus dashboards on Tableau public^11^. While Tableau facilitates powerful data visualization, the software is not open source and requires a license for use. To promote further development of CovidCounties and fully leverage the available data we have implemented our website using the commonly used *R* and *Rshiny* frameworks, and made all of our source code freely available on github (https://github.com/vivical/ButteLabCOVID).

CovidCounties represents an improvement over existing dashboards in terms of both scope and granularity. Existing COVID-19 dashboards generally focus either on county level data within a particular state (primarily at a static timepoint) or at the state level across the United States. We have developed an intuitive tool that facilitates temporal comparisons between all counties in the US. However, we are inherently limited by the availability of data. While CovidCounties’ estimation of ICU needs at the county level allows for higher resolution allocation of resources compared to the widely used state level model from IHME (https://covid19.healthdata.org/united-states-of-america), zip code level data would further improve the value of these estimations for resource allocation. States like Maryland^12^, Arizona^13^, and South Carolina^14^ and counties like Johnson County, Kansas^15^, San Diego County, California^16^, and King County, Washington^17^ have already made zip code level data available. However, there are many states and counties that are hesitant to provide data of this granularity due to concerns over privacy thus highlighting the challenge of balancing privacy with public good.

A limitation of CovidCounties is the inherent dependence on publicly available data. To date, most states and counties are primarily providing case and death data with an increasing number also providing hospitalization data and testing data. The increased availability of testing data opens new avenues to make inferences on the infection rate in the population and the improvement of model trajectories. As testing continues to ramp up, this will also allow for the evaluation of claims that there has been an under ascertainment of cases especially in the asymptomatic^18^, which can influence case rates. States and counties are continuously ramping up testing and this sudden availability of tests can artificially distort counts by attributing individuals who were infected previously to a later date due to an earlier shortage of tests. These numbers are further complicated by the wide variety of commercially available tests that rely on different technologies with varying sensitivity and specificity.

With its release, covidcounties.org represents a powerful open-source platform to empower non-data scientists to track the current trends of the COVID-19 pandemic at the county level to help facilitate policy and healthcare decisions which can help improve outcomes. We welcome volunteers (both technical and non-technical) to help us to further develop CovidCounties (https://covidcounties.org/buttelabcovid/www/volunteers.html).

## Methods

### Data sources

Data on state-wide and county-level counts were obtained from The New York Times^4^ via their *github* repository (https://github.com/nytimes/covid-19-data). County-wise population data were obtained from the US Census^19^ using the *R* package *tidycensus*^20^. Data on ICU bed availability per county was obtained from Kaiser Health News^21^.

As per The New York Times, cases and deaths reported from New York, Kings, Queens, Bronx and Richmond counties were assigned to New York City. Similarly, Cass, Clay, Jackson and Platte counties in Missouri were assigned to Kansas City. When a patient’s county of residence was unknown or pending many state departments reported these cases as coming from “unknown” counties. Cases reported from unknown counties were only included at the state level.

Data related to state-wide implementation of social-distancing policies were manually curated by web search and independently reviewed by a second author; disagreements were rare and resolved by discussion. Government websites were prioritized as sources of truth where feasible; otherwise, news reports covering state-wide proclamations were used. All citations are captured in the open data file accompanying this manuscript on Dryad^22^. These data were up to date and confirmed as of the date of data deposit: April 19, 2020.

Ground truth data used for validation were manually curated from the websites of multiple state departments of public health as well as Corona Data Scraper [https://coronadatascraper.com/], a commonly used resource for aggregating county-level tracking of COVID-19 over time. Citations of the validation data are included in the data file accompanying this manuscript on Dryad^22^.

Descriptive statistics on all datasets except that of the US Census and validation data are reported in Tables 1–3.

**Table 2:** Descriptive statistics on the Kaiser Health News data set.

*Kaiser Health News*		
*% of counties with non-missing ICU beds*	45.0%	
*Counties with most ICU beds*	Los Angeles, California (2,126)	Cook, Illinois (1,606)
*Counties with the most ICU beds per million*	Otero, Colorado (27,452)	Montour, Pennsylvania (2,303)
*Counties with the least ICU beds per million*	Wright, Minnesota (22)	Clinton, Michigan (25.2)

Data reported as of 8/20/2020. Counties with highest estimated ICU needs shows the ICU needs as a percentage based on the estimated number of ICU beds needed and KHN reported number of ICU beds.

**Table 3:** Descriptive statistics on the curated policy data set.

*Policy Data*		
*% of states with non-missing data for all 4 policies*	60.8%	
*First to declare state of emergency*	Washington (2/29/2020)	California (3/4/2020)
*First to close public schools*	Kentucky & Ohio (3/12/2020)	Delaware, Virginia & W Virginia (3/13/2020)
*First to declare shelter in place*	Arizona (3/11/2020)	California (3/19/2020)
*First to close restaurants and bars*	Ohio (3/15/2020)	12 states** on (3/16/2020)

Counties with highest estimated ICU needs shows the ICU needs as a percentage based on the estimated number of ICU beds needed and KHN reported number of ICU beds.

^**^CA, CT, DE, DC, KY, LA, MI, NJ, NY, PA, RI, WA.

### Doubling time

Doubling time was calculated for each state and county by taking the reciprocal of difference between the log (base 2) case counts corresponding to adjacent days, then applying the *R* function *loess* for smoothing. The input of this model required a minimum of 8 days of data where the minimum number of cases was greater than 10. Regularization was performed by replacing extreme doubling times (>500 days) with the average of the surrounding values.

### ICU bed occupancy model

We incorporated modified parameters related to rates of hospitalization and ICU admission from work previously published by Ferguson *et al*.^23^. Although simpler than other models, it fit publicly available county-level ICU bed data in California well and was easier to understand for the user than more complicated models proposed^9,24–26^. Our adapted model assumed an 8.26% rate of hospitalization among all new cases, a 29.64% rate of intensive care unit admission among hospitalized patients, and a 10-day average length of stay (time until discharge or death). The 29.64% average rate of ICU admission was estimated from empirical data on COVID-19 ICU admissions and total COVID-19 hospitalizations in obtained from state department of public health websites for each California county, and statewide for Minnesota, Idaho, Illinois, and Indiana from June 28th, 2020 to August 8th, 2020. The 8.26% average rate of hospitalization for positive cases was estimated from empirical data on the cumulative rate of hospitalization per 100,000 population from June 28th, 2020 to August 8th, 2020 across 14 states in COVIDNet^27^. The cumulative hospitalization rate was normalized to daily hospitalization rate for positive cases by normalizing by the cumulative positive cases per 100,000 population for the 14 states across the same time span.

To our knowledge there does not currently exist a high quality machine-readable standardized source of data for ICU and hospitalization rate parameter estimation. To ensure that our simple ICU model utilizes up to date parameters that reflect the current state of the pandemic, we will manually extract the relevant data each month and update these parameters. The parameters used in the live version of CovidCounties and all prior hospitalization and ICU rate parameter estimations will appear on the web application. This will serve as a log to reflect how time, testing, policy decisions, antivirals, and treatment options all affect the state of the pandemic.

Incident cases were chosen as the basis for the ICU bed occupancy model rather than incident deaths as they better track the dynamic changes in hospitalizations and ICU admissions. A model built around incident deaths has the advantage of being less dependent on testing capacity and thus the hospitalization parameters of such a model would be agnostic to the gamut of testing capabilities across states over the course of the pandemic. However, incident deaths are a lagging indicator of the current state of the pandemic as they result from infections from weeks prior and are therefore less informative about the immediate impacts of events such as the introduction or lifting of state mandates which are informative from a public policy standpoint.

### Web application development and deployment

**Fig. 4:**
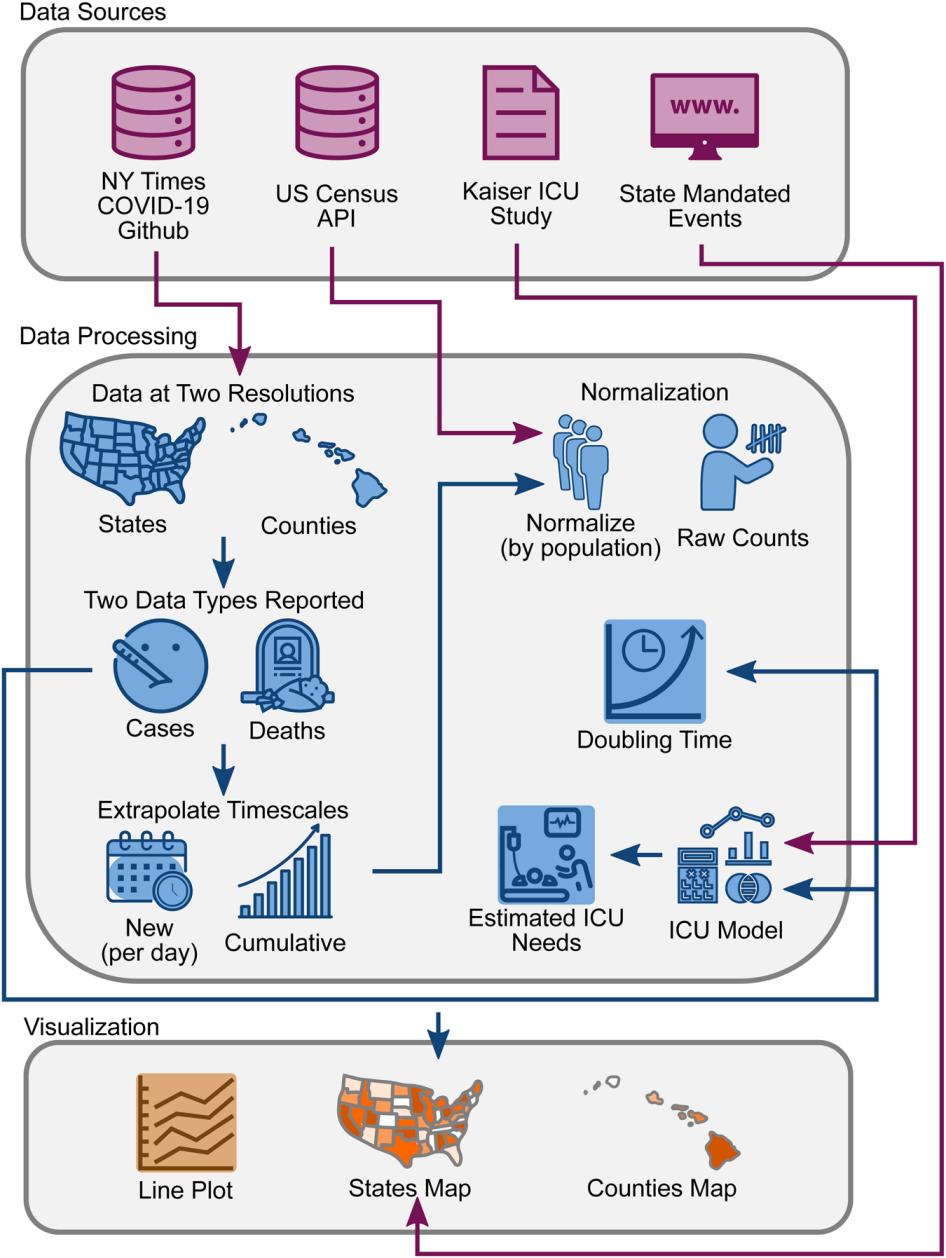
Database schematic. Source data was obtained from The New York Times, US Census Bureau, Kaiser Health News, and from a manual curation of state governmental websites and news outlets as described in *Methods*. Data was processed to reflect case and death counts at the level of states and counties. Functions were written to perform x- and y-axis rescaling, normalization by population, doubling time estimation, and ICU bed utilization. Results were depicted using interactive line plots and maps.

See Fig. 4 for an overall schematic of the web application. The source code was written in *R* (4.1.0)^28^ using the *shiny*^29^, *shinyjs*^30^, *tidyverse*^31^ and *plotly*^32^ packages. Software version control was achieved using Docker. The entire software code for the site is publicly available on *github* (https://github.com/vivical/ButteLabCOVID) and *dockerhub* (https://hub.docker.com/r/pupster90/covid_tracker). The web hosting was organized as a unified data share between all instances running *R shiny* code and controlled by a load balancer using an auto-scaling mechanism. The web environment is hosted by Amazon Web Services and is located at covidcounties.org. To confirm global accessibility of covidcounties.org, we used dareboost.com to perform loading speed tests from 14 cities across the globe using three different devices: Google Chrome via desktop, iPhone 6 s/7/8, and Samsung Galaxy S6. The results of the accessibility testing are on Dryad^22^.

## Usage Notes

A summary of the website features is available from the University of California, San Francisco [https://ucsf.app.box.com/v/Covid19Townhall041720]. A detailed tutorial illustrating use of the website is available on youtube.com (https://youtu.be/5OHDSpLv1kY).

## Data Availability

Curated data on the state-wide implementation of social-distancing policies and curated validation data are hosted on datadryad.com^22^.

## Code availability

The versions of software used have been detailed in the Methods section. The custom website source code is available on github (https://github.com/vivical/ButteLabCOVID). A version-controlled *Docker* container is also available on dockerhub (https://hub.docker.com/r/pupster90/covid_tracker).
